# Association between Education Attainment and Guideline-Directed Medication Therapy in Patients with Heart Failure and Reduced Ejection Fraction

**DOI:** 10.3390/jcm11144235

**Published:** 2022-07-21

**Authors:** Juan Long, Fanfang Zeng, Lili Wang, Honglei Zhao

**Affiliations:** Department of Cardiology, Fuwai Hospital Chinese Academy of Medical Science, Shenzhen 518001, China; ljdoctor@sohu.com (J.L.); zengfanfang123@tom.com (F.Z.); doctorllwang123@163.com (L.W.)

**Keywords:** heart failure, medications therapy, education attainment

## Abstract

**Objective:** The aim of the current study was to evaluate association of education attainment and guideline-directed medications therapy (GDMT) in patients with heart failure and reduced ejection fraction (HFrEF). **Method:** HFrEF patients were enrolled, and baseline characteristics were recorded. Based on highest educational attainment, patients were divided into low and high education attainment groups. Data on GDMT use at admission, discharge and follow-up were collected and between-group differences were evaluated. **Results:** A total of 336 patients were recruited, and 59.8% (n = 201) were defined as low education attainment. Patients with low education attainment were older and more likely to be female, obese and smokers. In addition, they had a higher prevalence of hypertension and valvular heart disease. Patients with low education attainment also had lower physical and mental component scores (PCS, 50.5 ± 6.4 vs. 56.3 ± 7.8), (MSC, 48.4 ± 6.0 vs. 54.7 ± 5.6) but higher serum NT-proBNP levels (1148.6 ± 233.4 vs. 1050.8 ± 205.6 pg/mL). Significant differences in GDMT use at admission, discharge and follow-up were observed. In the unadjusted model, high education attainment was associated with 2-fold odds of GDMT use at discharge. With adjustment for covariates, the high education attainment group remained significantly associated with being 22% more likely to receive GMDT at discharge. Similar findings were observed in associations between high education attainment and GDMT use at follow-up. After adjustment for PCS and MCS, high education attainment was still significantly associated with GDMT use at follow-up, with odds ratio of 1.13 and a 95% confidence interval of 1.08–1.28. **Conclusion:** HFrEF patients are under-treated. Education attainment is significantly associated with GDMT use at discharge and follow-up.

## 1. Introduction

Despite progress having been achieved in recent decades, heart failure (HF) remains a major cause of cardiovascular morbidity and mortality globally [1,2]. Notably, patients with HF have a poor quality of life (QoL) and high mortality risk in the first 5 years after symptoms occur [3,4]. Therefore, identifying the reasons for a poor prognosis in HF patients can provide scientific foundations to develop intervention strategies for HF patients.

Based on recommendations from HF guidelines [5], patients with HF and reduced ejection fraction (HFrEF) should receive a renin–angiotensin system (RAS) inhibitor, beta-blocker and mineralocorticoid receptor antagonist (MRA) therapy. However, data from Western populations indicate that the percentages of HFrEF patients that adhered to guideline-directed medications therapy (GDMT) was less than 50% [6,7], suggesting that there is huge room for improving HF management. Unfortunately, data on the adherence rate of GDMT among HFrEF patients in China are limited.

Interestingly, in the last two decades, numerous important studies have reported that education attainment was associated with cardiovascular outcomes in patients with coronary heart disease [8,9,10,11]. A low treatment and adherence rate of GDMT has been proposed to explain the association between education attainment and outcome [12,13,14].

With population ageing, the prevalence of HFrEF is projected to increase further in China [15,16,17]. In order to reduce the health and economic burden associated with HFrEF, we conducted a prospective study to evaluate whether education attainment was associated with GDMT use in HFrEF patients. We believe that findings from the current study can help us to better understand the knowledge gap in HFrEF management in China. Based on these findings, targeted interventions can be developed to improve HF management in the future.

## 2. Methods

### 2.1. Participants’ Enrollment

All subjects gave their informed consent for inclusion before they participated in the study. The study was conducted in accordance with the Declaration of Helsinki, and the protocol was approved by the Ethics Committee of Fuwai Hospital (FWHSZ20160902R1). Patients with a principal diagnosis of HFrEF at admission from January of 2017 to March of 2019 were screened, and the inclusion criteria were as follows: admission due to HF exacerbation, left ventricular ejection fraction (LVEF) <40% as assessed by echocardiography during index hospitalization, and discharge to home with stable status. The exclusion criteria were as follows: cardiogenic shock or required mechanical circulation support during hospitalization, death during index hospitalization, discharge with hospice, or patients who had contraindications to GDMT.

### 2.2. Data Collection

Data were extracted from electronic medical records by two independent physicians. Demographics included age and gender, and comorbidities included obesity (defined as body mass index ≥30 kg/m^2^), current cigarette smoking, hypertension, diabetes mellitus, dyslipidemia, atrial fibrillation, chronic kidney disease (CKD, defined as estimated glomerular filtration rate [eGFR] <60 mL/min/1.73 m^2^), coronary heart disease, valvular heart disease, dilated cardiomyopathy and ischemic stroke. Quality of life (QoL) was assessed by Short Form-12 physical and mental component score (PCS and MCS) [18]. Patients were categorized into low and high education attainment groups using college degree as the cutoff. Laboratory parameters at admission were also extracted from electronic medical record. Medications used at admission were recorded and reconciled with patients’ family members. Medications used at discharge and at one month follow-up after discharge were assessed.

### 2.3. Study Objectives

The objectives of this study were to evaluate whether education attainment was associated with GDMT prescription at discharge and GDMT adherence at one month follow-up after discharge in HFrEF patients.

### 2.4. Statistical Analysis

Continuous variables were presented as mean ± standard deviation (SD) and compared by Student’s *t* test; categorical variables were presented as number and proportions and compared by the chi-square or Fisher’s exact test as appropriate. Multivariate regression analysis was performed to evaluate the association between education attainment and GDMT use at discharge and adherence at one month’s follow-up, and the low education attainment group was served as the reference group. Specifically, GDMT in this study was referred to as a RAS inhibitor, beta-blocker, or MRA therapy. Statistical analyses were computed using SPSS 17.0 (SPSS Inc., Chicago, IL, USA,). All statistical tests were two-sided and considered statistically significant when *p* < 0.05.

## 3. Results

### 3.1. Comparisons of Baseline Characteristics by Education Attainment

**Figure 1:**
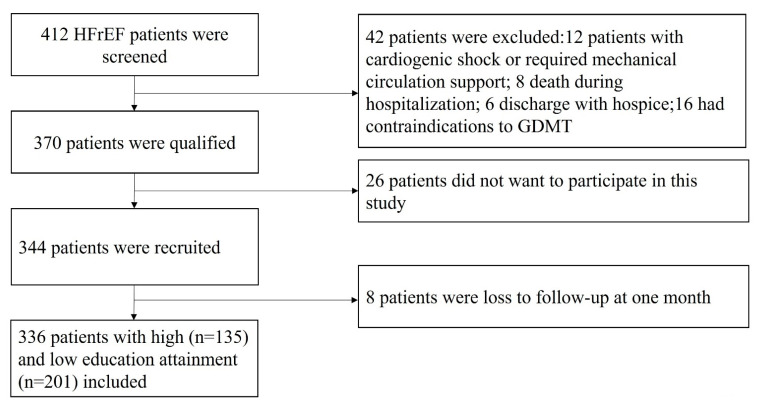
Study flowchart.

In this study, a total of 336 patients were included in the final analysis and 59.8% (n = 201) had an educational level lower than a college degree (study flowchart was presented in Figure 1). Baseline characteristics were compared by education attainment. As shown in Table 1, compared to patients with a high education attainment, those with low education attainment were older (51.6 ± 10.7 vs. 44.5 ± 11.6 years) and more likely to be female (61.2% vs. 51.9%), obese (28.9% vs. 25.2%) and smokers (29.9% vs. 22.2%). In addition, they had higher prevalence of hypertension (54.7% vs. 44.4%) and valvular heart disease (43.8% vs. 35.6%). Patients with low education attainment had a higher heart rate (84 ± 13 vs. 78 ± 12 beat per minute), and serum level of N-terminal pro-B natriuretic peptide (NT-proBNP,1148.6 ± 233.4 vs. 1050.8 ± 205.6 pg/mL); while those with high education attainment had lower PCS (50.5 ± 6.4 vs. 56.3 ± 7.8) and MSC (48.4 ± 6.0 vs. 54.7 ± 5.6). At discharge, PCS (61.4 ± 7.2 vs. 66.5 ± 8.1) and MCS (54.7 ± 7.2 vs. 62.3 ± 6.4) were improved in both groups, while there were still significant between-group differences. At follow-up, PCS and MCS were decreased when compared to discharge, and the high education attainment group still had higher PCS (55.6 ± 7.0 vs. 60.1 ± 7.5) and MCS (51.3 ± 6.6 vs. 56.2 ± 6.0).

### 3.2. Comparisons GDMT Used by Education Attainment

As presented in Table 2, at admission, the percentages of patients receiving GDMT were extremely low in both groups, and between-group differences in beta-blocker use were observed (47.3% vs. 56.3%). At discharge, the use of GDMT (RAS inhibitor, beta-blocker, MRA) and furosemide were increased in both groups. Significant differences in RAS inhibitor (69.7% vs. 87.4%) and beta-blocker (56.7% vs. 68.1%) use were observed. At one month’s follow-up, RAS inhibitor, beta-blocker and furosemide use significantly decreased in the low education attainment group; and beta-blocker and furosemide use significantly decreased in the high education attainment group. Between-group differences in RAS inhibitor (63.7% vs. 85.2%) and beta-blocker (49.3% vs. 59.3%) use were persistent at one-month follow-up.

### 3.3. Associations between Education Attainment and GDMT Use at Discharge and Follow-up

As presented in Table 3, in the unadjusted model, high education attainment was associated with approximately 2-fold odds of GDMT use at discharge. With a stepwise adjustment for potential covariates, the odds gradually decreased. After adjustment for PCS and MCS, high education attainment remained significant.

Similar findings were observed in the associations between high education attainment and GDMT use at follow-up. After adjustment for PCS and MCS, high education attainment was still significantly associated with GDMT use at follow-up, with an odds ratio of 1.13 and 95% confidence interval of 1.08–1.28.

## 4. Discussion

To our knowledge, this is one of the first studies to evaluate GDMT use in HFrEF patients in China. For the first time, we evaluated whether education attainment is associated with GDMT use at discharge and follow-up. Our study suggests that GDMT use was extremely low in HFrEF patients in China. Although GDMT use at discharge increased, a large proportion of patients discontinued GDMT use after one month’s follow-up. Compared to patients with high education attainment, those with low education attainment had lower use of GDMT. Together, these findings suggest that, despite GDMT being demonstrated to improve the prognosis in HFrEF patients, GDMT remains under-use in China. Future studies are needed to investigate the barriers in implementing GDMT in daily clinical practice, particularly for those with low education attainment in China.

Heart failure is a major public health issue globally [7,19]. Randomized clinical trials demonstrated that a RAS inhibitor, beta-blocker and MRA were beneficial for reducing the risk of hospitalization and mortality for HFrEF patients. Nonetheless, epidemiological studies on Western populations and post hoc analyses of clinical trials showed that a large proportion of HFrEF patients received suboptimal medications therapy [6,20]. The reasons for this are likely multifactorial. For example, it could be due to problems that occur during the transition of care between inpatient and outpatient clinical encounters [21]. Some studies suggested that a proportion of physicians were unwilling to alter their prescriptions despite the fact that their patients had worsening conditions [6,22]. HF patients with low blood pressure or severe renal dysfunction were less likely to receive GDMT or adhere to GDMT. In addition, patients without health insurance were also more likely to discontinue GMDT [11,12].

Consistent with prior reports from Western populations, our findings suggest that GDMT use in Chinese HFrEF patients was extremely low. We were unsure of the underlying reasons. However, the low use of GDMT in this study should not be caused by contraindications since we recruited patients who were eligible to GDMT. Since prior studies suggested that education attainment was associated with medication use in patients with CHD, we hypothesized that education attainment might be associated with GDMT use in HFrEF patients. Besides RAS inhibitors, beta-blockers and MRA, diuretic is also important for HF management. We found that both at admission, discharge and follow-up, the use of furosemide was high in both groups, suggesting that most of these patients might still have clinical symptoms and signs.

Interestingly, we observed that there were significant differences in comorbidities and other baseline characteristics between high and low education attainment groups. In general, compared to the high education attainment group, patients in the low education attainment group had more comorbidities and poorer QoL, which was also observed in patients with CHD in the US and Europe. At admission, both high and low education attainment patients were at low GDMT use, suggesting that there was huge gap in implementing GDMT for HFrEF patients in China, regardless of their education attainment. At discharge, GDMT use was increased. However, as presented in Table 2, the percentages of patients who received GDMT (RAS inhibitor, beta-blocker and MRA combined) were only 20.9% and 27.4%, respectively. Furthermore, after one month’s follow-up, the percentage of patients adhering to GDMT use significantly decreased, especially in the low education attainment group. These findings suggest that the management of HFrEF patients were poor in before, during, and after hospitalization. Further studies are needed to investigate the reasons for the low use of GDMT in HFrEF patients from China.

In order to evaluate the potential factors mediating education attainment and GDMT use, we performed a stepwise regression analysis. As presented in Table 3, it is noted that, after adjusting for QoL and as indicated by PCS and MCS (Model 4), the odds ratio for the high education attainment group to receive GDMT was reduced by 23% at discharge and 22% at follow-up, respectively. These findings imply that the association between education attainment and GDMT use in HFrEF patients might be predominantly mediated by QoL. After adjustment for potential covariates, high education attainment was still significantly associated with higher odds of GDMT use, suggesting that low education attainment might be a potential risk factor for low GDMT use. Future studies are needed to address these health disparities in HFrEF management incurred by education attainment.

The strategies to address the low adherence rate of GDMT could focus on the following aspects. Firstly, education on the importance of adhering to GDMT should be applied to both the patients and their relatives before discharge. Secondly, regular follow-up after discharge should be performed. Thirdly, improvement in the accessibility of GMDT for people who live in remote areas is critical.

There are some limitations of this study. Firstly, this was an observational study, and no causal relationship could be determined. Secondly, this was a single-center study and multiple-center studies are needed to corroborate our findings. Thirdly, since we had only followed up patients for one month, whether the association between education attainment and GDMT use persists after long-term follow-up is unknown. Fourthly, we did not collect data on SGLT2i and ARNI use; therefore, we were unable to evaluate the uptake of these two novel medications in Chinese HFrEF patients. Last but not least, although we extensively adjusted for potential covariates, undetected and unmeasured covariates could still exist that influenced the associations between education attainment and GDMT use.

## 5. Conclusions

In conclusion, our findings suggest that HFrEF patients in China are under-treated, especially those with low education attainment. Education attainment is significantly associated with GDMT use at discharge and at follow-up. Future studies are needed to investigate the reasons for low GDMT use in HFrEF patients from China.

## Figures and Tables

**Table 1:** Comparisons of baseline characteristics by education attainment.

Variables	Low Education Attainment (n = 201)	High Education Attainment (n = 135)
Age (years)	51.6 ± 10.7 *	44.5 ± 11.6
Female, n (%)	123 (61.2) *	70 (51.9)
Obese, n (%)	58 (28.9) *	34 (25.2)
Systolic blood pressure (mm Hg)	124 ± 16	125 ± 15
Diastolic blood pressure (mm Hg)	79 ± 10	77 ± 10
Heart rate (beat per minute)	84 ± 13 *	78 ± 12
Current smoker, n (%)	60 (29.9) *	30 (22.2)
Diabetes mellitus, n (%)	40 (19.9)	25 (18.5)
Hypertension, n (%)	110 (54.7) *	60 (44.4)
Dyslipidemia, n (%)	62 (30.8)	42 (31.1)
Atrial fibrillation, n (%)	95 (47.3)	64 (47.4)
Chronic kidney disease, n (%)	28 (13.9)	18 (13.3)
Coronary heart disease, n (%)	64 (31.8)	44 (32.6)
Valvular heart disease, n (%)	88 (43.8) *	48 (35.6)
Idiopathic dilated cardiomyopathy	40 (19.9)	28 (20.7)
Ischemic stroke, n (%)	53 (26.4)	37 (27.4)
Physical component score	50.5 ± 6.4 *	56.3 ± 7.8
Mental component score	48.4 ± 6.0 *	54.7 ± 5.6
Glycated hemoglobin A1c (%)	6.6 ± 1.2	6.5 ± 1.1
Total cholesterol (mmol/L)	5.0 ± 0.9	5.0 ± 1.0
Sodium (mEq/L)	134.2 ± 4.6	133.6 ± 4.2
Potassium (mEq/L)	3.8 ± 0.9	3.9 ± 0.7
Creatinine (umol/L)	66.5 ± 21.8	67.8 ± 20.7
eGFR (ml/min/1.73 m^2^)	74.5 ± 15.8	75.8 ± 16.6
NT-proBNP (pg/mL)	1148.6 ± 233.4 *	1050.8 ± 205.6
LVEF (%)	32.5 ± 6.7	33.8 ± 5.5

eGFR, estimated glomerular filtration rate; NT-proBNP, N-terminal pro-B natriuretic peptide; LVEF, left ventricular ejection fraction; * *p* < 0.05 versus high education attainment group.

**Table 2:** Comparison medications used by education attainment.

Medications	Low Education Attainment (n = 201)	High Education Attainment (n = 135)
**At admission**		
ACEi/ARB	137 (68.2)	95 (70.4)
Beta-blocker, n (%)	95 (47.3) *	76 (56.3)
MRA, n (%)	50 (24.9)	33 (24.4)
Combined	30 (14.9)	20 (14.8)
Furosemide, n (%)	148 (73.6)	101 (74.8)
Hydrochlorothiazide, n (%)	66 (32.8)	42 (31.1)
Digoxin, n (%)	87 (43.3)	62 (45.9)
Anti-platelet, n (%)	68 (33.8)	47 (34.8)
Statins, n (%)	54 (26.9)	36 (26.7)
Anti-diabetes, n (%)	36 (17.9)	23 (17)
**At discharge**		
ACEi/ARB	140 (69.7) *	118 (87.4)
Beta-blocker, n (%)	114 (56.7) *	92 (68.1)
MRA, n (%)	53 (26.4)	38 (28.1)
Combined, n (%)	42 (20.9) *	37 (27.4)
Furosemide, n (%)	196 (97.5)	130 (96.3)
Hydrochlorothiazide, n (%)	60 (29.9)	38 (28.1)
Digoxin	100 (49.8)	70 (51.9)
Anti-platelet	67 (33.3)	47 (34.8)
Statins	54 (26.9)	34 (25.2)
Anti-diabetes	36 (17.9)	24 (17.8)
**At one-month follow-up**		
ACEi/ARB	128 (63.7) *	115 (85.2)
Beta-blocker, n (%)	99 (49.3) *	80 (59.3)
MRA, n (%)	51 (25.4)	37 (27.4)
Combined, n (%)	36 (16.7) *	33 (24.4)
Furosemide, n (%)	182 (90.5)	121 (89.6)
Hydrochlorothiazide, n (%)	52 (25.9)	33 (24.4)
Digoxin	100 (49.8)	70 (51.9)
Anti-platelet	67 (33.3)	46 (34.1)
Statins	54 (26.9)	34 (25.2)
Anti-diabetes	36 (17.9)	23 (17)

ACEi/ARB, angiotensin-converting enzyme inhibitor/angiotensin receptor blocker; MRA, mineralocorticoid receptor antagonist; combined indicates ACEi/ARB + beta-blocker + MRA * *p* < 0.05 versus high education attainment group.

**Table 3:** Associations between education attainment and GDMT use at discharge and follow-up.

Models	Odds Ratio	95% Confidence Interval
**GDMT prescription at discharge**		
Unadjusted	1.96	1.65–2.48
Model 1	1.78	1.51–2.17
Model 2	1.64	1.43–1.86
Model 3	1.45	1.29–1.63
Model 4	1.22	1.14–1.39
**GDMT use at follow-up**		
Unadjusted	1.87	1.70–2.27
Model 1	1.72	1.50–2.03
Model 2	1.53	1.25–1.76
Model 3	1.35	1.14–1.50
Model 4	1.13	1.08–1.28

GDMT prescription indicates ACEi/ARB + beta-blocker + MRA use; **Model 1**: adjusted for age; **Model 2**: further adjusted for obesity, smoking, diabetes mellitus, hypertension, dyslipidemia, atrial fibrillation, chronic kidney disease, ischemic stroke; **Model 3**: further adjusted for coronary heart disease, valvular heart disease, idiopathic dilated cardiomyopathy; **Model 4**: further adjusted for physical component score and mental component score.

## Data Availability

Data is available upon reasonable request from the corresponding author.
